# Metal-Enhanced Fluorescent Carbon Quantum Dots via One-Pot Solid State Synthesis for Cell Imaging

**DOI:** 10.1021/acsabm.3c00040

**Published:** 2023-04-26

**Authors:** Volkan Can, Bugra Onat, Elif Sümeyye Cirit, Fikrettin Sahin, Zeliha Cansu Canbek Ozdil

**Affiliations:** † Department of Genetics and Bioengineering, Yeditepe University, Istanbul 34755, Turkey; ‡ Department of Materials Science and Nanotechnology Engineering, Yeditepe University, Istanbul 34755, Turkey

**Keywords:** carbon quantum dot, gold nanoparticle, metal-enhanced fluorescence, bioimaging, solid-state synthesis

## Abstract

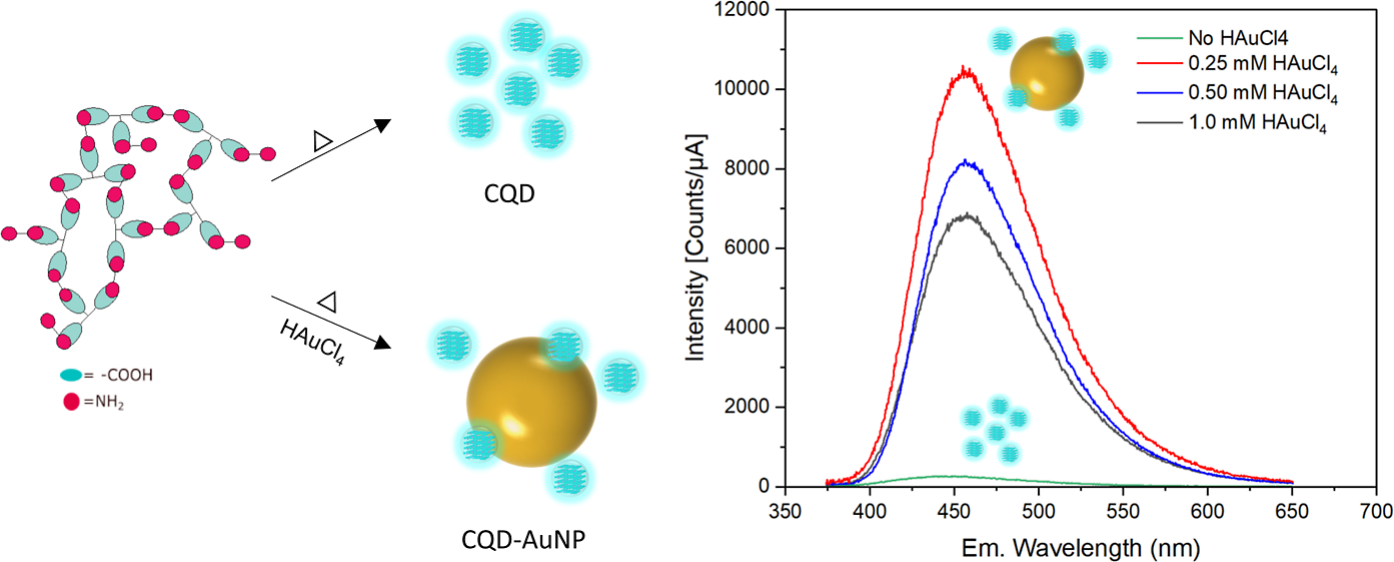

In this study, a facile one-pot solid-state synthesis method is developed to shed light on the metal-enhanced fluorescence (MEF) effect in carbon quantum dots (CQDs) and gold nanoparticles (AuNPs) hybrid materials. This is one of the few studies on the solid-state synthesis of N-doped CQDs/gold hybrid nanomaterials. We have conducted various sets of experiments to reveal the role of individual reagents during the nucleation and growth of nanoparticles. We have demonstrated that the addition of a small amount of gold salt illustrates a paramount effect (10^3^-fold) in photoluminescence intensity. This effect is ascribed to MEF, which is caused due to interactions between the excited-state fluorophores and the free surface electrons of metal nanoparticles. It is interesting to note that a further increase of gold yields fluorescence quenching due to a large number of formed AuNPs causing fluorescence resonance energy transfer. By adjusting the volume ratio of gold salt and CD precursors, it is possible to obtain the CQDs–AuNPs hybrid with the highest fluorescence, which produces extensive visible light under 460 nm excitation. Synthesized materials have been successfully used for imaging human dermal fibroblasts and A549 lung epithelial cells. The dose-dependent cytotoxicity studies reveal that the hybrid structures do not have cytotoxicity.

## 1 Introduction

Nanotechnology has enormous potential as a remedial approach for targeted cell imaging with the help of highly functional fluorescent nanomaterials bearing exceptional emission properties and high surface functionalities to assist selectivity toward cells of interest.^1−3^ Antibody-fluorescent nanoparticle conjugates are promising systems providing high imaging possibilities with increased dependability, sensitivity, and specificity.^4^

Fluorescent carbon quantum dots (CQDs) are quasi-spherical nanoparticles used in various biomedical applications, such as sensing, bioimaging, or drug delivery, due to their exceptional water solubility properties as well as their high biocompatibility and chemical inertness.^5−7^ Thanks to their small size (<10 nm), CQDs have photoluminescence (PL) emission in the visible region. Depending on the preparation method, the surface of the nanomaterial may contain alternative functional groups, such as amino, hydroxy, or carboxy, which strongly influence the fluorescence quantum yield, color, and quenching mechanisms of the CQDs.^8^ Additionally, heteroatom doping is known as an effective strategy to enhance the fluorescence intensity of CDs.^9^ Even though the exact mechanism behind PL enhancement via doping is not fully understood, proposed mechanisms such as enhanced electron transfer caused by the presence of nitrogen atoms in the carbon matrix or the creation of new energy levels with nitrogen in the carbon core leading to increased PL are found in the literature.^10,11^ On the whole, the utilization of ethylenediamine as a nitrogen source for nitrogen doping in carbon dots leads to an improvement in the PL quantum yield and stability, thereby enhancing the potential practical applications of carbon dots. Due to the quantum confinement effect, the size of the nanoparticle also strongly alters the optical properties of the material; thus, it is possible to tune their properties easily for different kind of applications.^12^

Hybrid nanomaterials based on fluorescent and metallic constituents, combining multiple imaging modalities and high specificity added up into a single domain, have recently emerged as a new frontier in molecular oncologic imaging.^3,6^ Nanomaterials that possess the intrinsic properties of both components are promising new-generation contrast agents, providing multimodal imaging possibilities. When the CQDs are coupled with metallic magnetic nanoparticles, as in the work of Wang et al.,^13^ to generate hybrid 1D nanoparticle chains possessing magnetic and fluorescence properties, the advanced material shows excellent MRI contrast agent properties as well as fluorescence imaging possibilities. The material is based on magnetic iron oxide nanocrystals clustered in the core section, while CQDs comprise a shell around the magnetic substituent. Liu et al. demonstrated that metal–CQDs hybrids can also be used as catalysts since the material illustrates paramount photocatalytic activity for the selective oxidation of cyclohexane to cyclohexanone and cyclohexanol.^14^

In the conjunction of CQDs with plasmonic metal nanoparticles, it is known that due to the excitation coupling between the CQDs and the metal nanoparticle, the PL intensity of the hybrid can be enhanced or quenched depending on the overlap between the emission and absorption bands of each constituent.^5,6,15,16^ Metal-enhanced fluorescence (MEF) is mostly due to increased resonance energy transfer caused by the interactions between the excited-state fluorophores and the free surface electrons of metal nanoparticles.^1,15−18^ On the contrary, fluorescence quenching based on fluorescence resonance energy transfer (FRET) between highly absorbing metal nanoparticles and fluorescent nanomaterials is caused by overlapping between the emission spectra of the donor and acceptor metal nanoparticles.^19,20^

Among metallic nanoparticles, due to unique optical properties at the nanoscale, gold is becoming more and more popular in the field of medical imaging. This is because hybrid systems containing gold substituents generate a specific signal called surface plasmon resonance (SPR) in the ultraviolet and visible region bands as a result of electromagnetic excitation. Basically, because of the interaction between the gold constituent and specific biomarkers used for disease diagnosis, a change in the peripheral refractive index can be observed in the visible region through SPR. Various fabrication methods for the production of CQDs–AuNPs hybrids are presented in the literature (Table 1). Most of these techniques are based on ex situ hybrid material production, meaning both constituents are produced via separate complex production methods and mixed to obtain the fluorescence/plasmonic hybrid material.^21,22^ Between those, only a few studies have focused on solid-state synthesis.^23^

**Table 1:** Comparison of the Production Methods of CQDs–AuNPs Spherical Hybrids

method					
CDs synthesis	AuNP synthesis	precursor	reaction media	reaction time and temperature (°C)	refs
hydrothermal	wet chemical reduction via PEG	*p*-phenylenediamine (pPD) and nitric acid for CQD	aqueous	4 h/180 °C	(Pawar et al., 2022)
		HAuCl_4_·3H_2_O for AuNP			
microwave	wet chemical reduction via trisodium citrate dihydrate	citric acid and 2,2′-(ethylene-dioxy)bis(ethylamine) for CQD	aqueous	2.5 min	(Shi et al., 2014)
		HAuCl_4_·3H_2_O for AuNP			
electrochemical	wet chemical reduction via trisodium citrate dihydrate	ethanolamine for CQD	aqueous	5 h/RT	(Deng et al., 2015)
		HAuCl_4_·3H_2_O for AuNP			
microwave	wet chemical reduction via as-synthesized CQD	ascorbic acid for CQD	aqueous	30 min/130 °C	(Mehta et al., 2018)
		HAuCl_4_·3H_2_O for AuNP			
in situ mixing	wet chemical reduction via cetylpyridinium chloride monohydrate (CPC)	CPC for CQD	aqueous	1 h/RT	(Zhu et al., 2020)
		HAuCl_4_·3H_2_O for AuNP			

In this study, we report the effect of reaction parameters on the optical properties of N-doped CQDs–AuNPs hybrid materials obtained via one-pot solid synthesis methods. To be specific, the effect of CQDs and gold precursor concentrations on fluorescence intensity was studied, as was the effect of carbon dot precursor concentration on the PL emission yield and whether the system complies with the plasmonic resonant effect or FRET mechanism. A549 lung epithelial cell line is a well-known non-small cell lung cancer cell line, and the human dermal fibroblast (HDF) cell line is a well-known general control cell line that has been used for comparing the toxicity and proliferation capacity of the CQDs–AuNPs in cancer and healthy cell lines. To test the efficiency of the fluorescence enhancement, uptake, and bioimaging capacity of the material, the same cell lines have been used as a model bioimaging system to test the efficiency of the fluorescence enhancement of the material.

## 2 Materials and Methods

### 2.1 Materials

Tetrachloroauric(III) acid trihydrate (HAuCl_4_·3H_2_O), citric acid (CA), sodium tricitrate dihydrate, and ethylene diamine (EDA) were obtained from Sigma-Aldrich and used without further purification. Prior to experiments, all glassware was cleaned with aqua regia and washed with distilled water. Adenocarcinoma human alveolar basal epithelial cells (A549, CCL-185, ATCC) and HDF cells (HDFa, PCS-201-012, ATCC) cell lines were purchased from the American Type Culture Collection.

### 2.2 Methods

#### 2.2.1 Hybrid Nanomaterial Preparation via Pre-synthesized AuNPs

Gold nanoparticles (AuNPs) that are used during the study originated from the work of Sivaraman et al.^24^ In this method, AuNPs are prepared by reversing the reactant addition order in the standard Turkevich method^25^ to obtain smaller-sized nanoparticles and have better control over the size distribution. In this method, 0.25 mL of 25.4 mM HAuCl_4_ is mixed with 24.75 mL of 5.2 mM boiling citrate solution (*T* ∼ 90 °C). The reaction terminates after 250 s. The size of the particles is measured immediately after the preparation by dynamic light scattering (DLS), and the measurement was repeated before the preparation of the CQDs–AuNPs hybrid production to ensure that the average diameter remains constant.

CQDs–AuNPs hybrid preparation is realized via the addition of 50, 250, and 500 μL of pre-synthesized AuNPs into a 10 mL solution of 0.498 M EDA and 0.546 M CA. In the following part, the mixtures are dried at 65 °C overnight, heated up to 160 °C for 2 h, and dissolved in 10 mL of Milli-Q water.

#### 2.2.2 Hybrid Nanomaterial Preparation via Gold Salt

During the synthesis of the hybrid, CA is used both as a carbon precursor during the formation of CQDs and as a reducing/capping agent during the production of AuNPs. In a typical synthesis, 0.498 M EDA and 0.546 M CA are dissolved in 10 mL of Milli-Q water. In the next step, 25, 50, and 100 μL of 0.1 M HAuCl_4_ salt is added rapidly under continuous stirring. After that, the solutions are dried in an oven at 65 °C overnight to obtain a thin solid film. The obtained dry-form samples are heated to 160 °C for 2 h and redissolved in 10 mL of Milli-Q water.

#### 2.2.3 Characterization of Nanomaterials

Produced nanomaterials are characterized via multiple techniques. Absorption characteristics were analyzed via UV–vis spectroscopy. The spectra of final particles are measured in a 10 mm—Helma cell by using the PerkinElmer LAMBDA 25 Series UV–vis spectrophotometer. The obtained data are analyzed with UV WinLab software. Morphological characterization of the nanoparticles is realized via transmission electron microscopy (TEM). The observations are carried out by a JEOL JEM 2100 Plus microscope equipped with a Gatan US4000 CCD camera operating at 200 kV. The size distribution of nanoparticles was monitored using DLS. The analysis is carried out using a Zetasizer (Malvern Instruments) at 25 °C. The volume size distribution and the polydispersity index were obtained from the autocorrelation function using the general-purpose mode for all analytes. The fluorescence spectroscopy analysis is realized via a HORIBA DUETTA absorption and fluorescence spectrophotometer at variable excitation wavelengths. The spectra are collected within the range of 365–600 nm.

#### 2.2.4 Cell Culture and Viability Assessment

Adenocarcinoma human alveolar basal epithelial A549 (CCL-185 ATCC) and human dermal fibroblast HDF (HDFa ATCC) cell lines are grown in DMEM with high glucose supplemented with 10 % fatal bovine serum, 1 % penicillin/streptomycin, and amphotericin B solution. The cells are maintained at 37 °C in a 5 % CO_2_-containing incubator equipped with a humidified atmosphere. The cells are passaged when they reach 80 % confluences to maintain growth with the help of a trypsin-EDTA solution.

#### 2.2.5 Cell Viability Assay

The MTS metabolic catalytic assay is used to determine cell viability after exposure to the hybrid nanomaterial. A549 and HDF cell lines are seeded in different 96 well plates within the replicates at 5000 cells/plate in a total volume of 150 μL, and the plates are incubated at 37 °C. Afterward, nanomaterials were injected rapidly to elucidate the potential cytotoxicity of the probes for 24, 48, and 72 h. After the treatment, the old medium is aspirated with a fresh solution of 4.5 g/L d-glucose in PBS mixed with the MTS reagent. After 1 h of incubation, the absorbance values of each well are determined by using an ELISA plate reader (BioTek Instruments) at 490 nm.

#### 2.2.6 Cellular Apoptosis Detection

To understand the apoptotic effect of CQDs–AuNPs, the eBioscience Annexin V Apoptosis Detection Kit (Invitrogen catalog number BMS500FI-300) is used. Briefly, A549 and HDF cells are seeded in different 6 well plates with a density of 2.5 × 10^5^ in 2 mL of fresh complete medium. The plates are incubated at 37 °C for 24 h to ensure the attachment of the cells. The cells are treated with different amounts of CQDs–AuNPs for 24 h. Then, the cells are taken with trypsin EDTA and washed with 1× PBS. After each wash, the cells are centrifuged at 1500 rpm or 300 G for 5 min at room temperature. After that, the cells are suspended with 200 μL 1× binding buffer, which contains 5 μL of annexin-v-FITC antibody. The cells were incubated for 20 min at RT in the dark and washed twice with 1× binding buffer. Before the flow cytometry reading, 3 μL of the propidium iodide (PI) solution is added to each sample. The results are analyzed with Millipore Guava EasyCyte. To understand the apoptotic capacity, 10,000 cells are read at each trial.

#### 2.2.7 Quantification of Cellular Uptake of Nanoparticles

A549 and HDF cells are seeded in 6 well plates with a density of 2.5 × 10^5^ in 2 mL of fresh complete medium. The plates are incubated at 37 °C for 24 h to ensure the attachment of the cells. The cells are treated with different amounts of CQDs–AuNPs for 24 h. Afterward, the wells are washed with PBS to remove the excess nanoparticles from the environment. 5 min of RT incubation with ice-cold absolute methanol is used as a fixative reagent. 10 μg/mL PI is applied to the cells. After 10 min of treatment, the plates are washed with PBS, and then the coverslips are attached by using glycerol (cryo glue). The cells are analyzed with a ZEISS confocal laser scanning microscope equipped with a filter of DAPI and RED. All images are recorded with 20× objectives and analyzed with Zen Software.

## 3 Results and Discussion

In a typical synthesis of CQDs–AuNPs hybrid materials, hydrothermal and wet chemical methods have been traditionally employed to obtain high-yield nanomaterials, followed by mixing these nanoparticles in a separate container to obtain the hybrid.

**Scheme 1:**
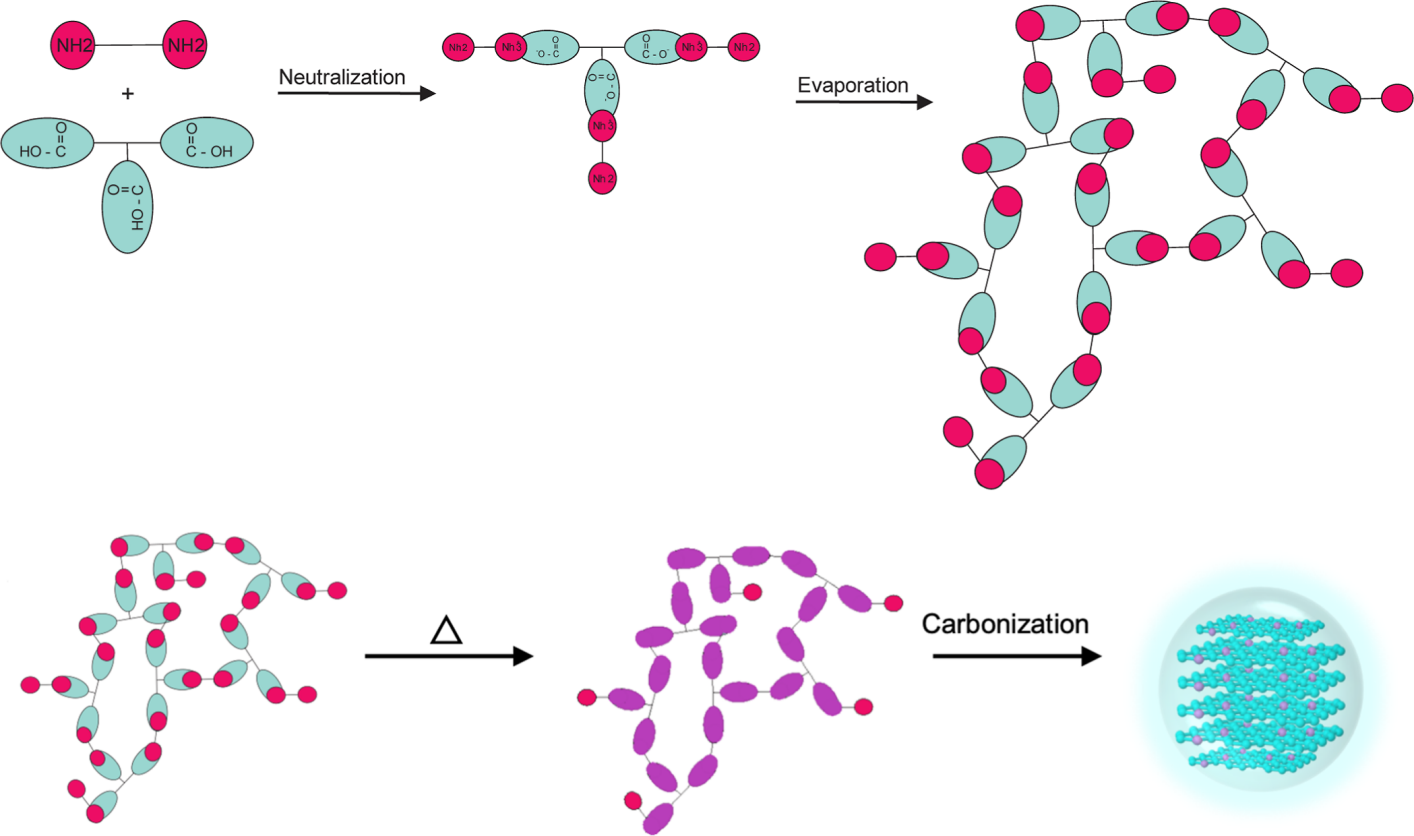
Formation of CA–EDA Ion Pair Crystals via Neutralization Followed by Solvent Evaporation, High-Temperature Reaction, and N-doped C-dot Formation

The solid-state synthesis method used in this study is inspired by the work of Tomaz et al.,^26^ where a solid-state reaction between an organic acid and an amine is shown. In their interesting study, they took advantage of oriented conjugate salts with a certain order, produced by the neutralization reaction between ethylenediamine tetraacetic acid (EDTA) and EDA at different ratios and solvent evaporation. Conjugate salts of the acid–base reaction are crystallized in a certain order with solvent evaporation. After that, the reaction is conducted at high temperatures. They concluded that the reaction resulted in hyperbranched polyamides with dendritic structures. In this study, we have utilized an analogous system with CA and EDA (Scheme 1) and conducted the reaction at high temperatures (160 °C) for 2 h with and without the presence of the AuNP precursor. We have shown that the resultant structures have fluorescent carbon dot properties, and the presence of AuNPs has a significant amplification effect on the emission intensity of the resultant structure.

Due to limited studies on the solid-state synthesis of hybrid materials, our initial attempt was to use pre-synthesized AuNPs. In this method, various concentrations of AuNPs obtained via the reverse Turkevich^24^ method were mixed with CQD precursors at 65 °C overnight and heated up to 160 °C.

**Figure 1:**
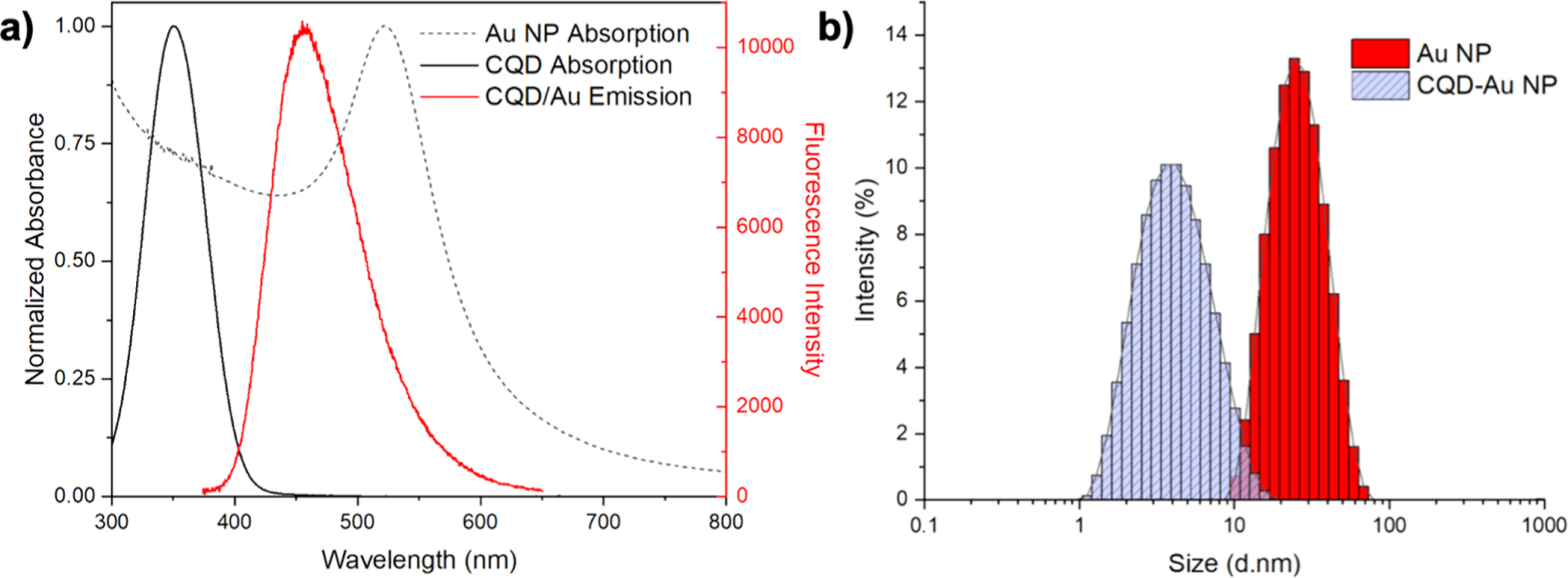
(a) UV–vis absorption spectrum of AuNPs and CQDs with the PL emission spectrum of CQDs–AuNPs hybrid and (b) corresponding DLS histograms of AuNPs and CQDs–AuNPs hybrids.

The normalized UV–vis absorption spectrum of bare CQDs and AuNPs, obtained via solid-state synthesis, is illustrated in Figure 1a with the CQDs–AuNPs emission spectrum. The bare CQD absorbs light around 351 nm, whereas AuNPs show absorption maxima around 524 nm. There is a large overlap between the CQDs and AuNPs (SPR) absorption bands.

The DLS histogram of AuNPs indicates an average size of 23 ± 7 nm (Figure 1b). In the hybrids where pre-synthesized AuNPs were used, we observed high quantities of black precipitates and failed to have stable colloidal suspension.

**Figure 2:**
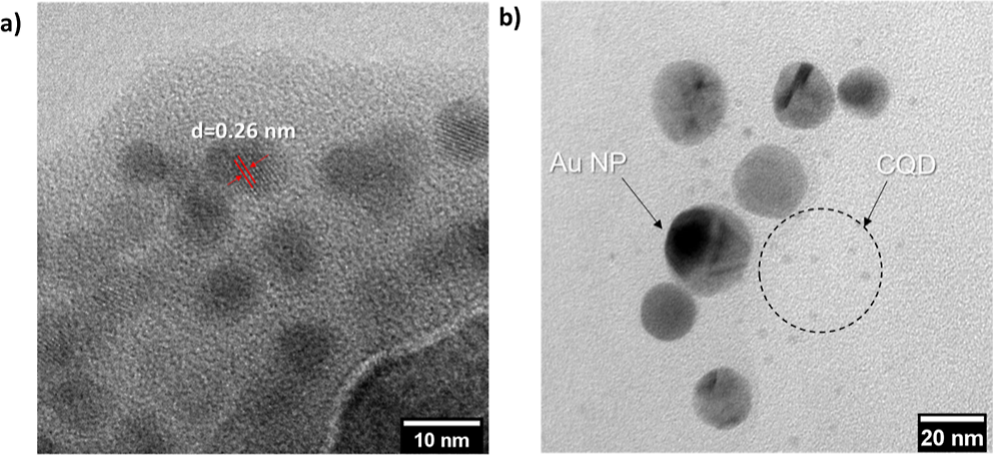
Representative TEM micrographs of (a) CQDs and (b) CQDs–AuNPs hybrid.

Separately, in one-pot solid-state synthesis, where the pre-synthesized AuNP solution was replaced with HAuCl_4_·3H_2_O salt to synthesize metal nanoparticles simultaneously with CQD, no black precipitate was observed. The average size of the CQDs–AuNPs hybrid is found to be around 4 ± 3 nm indicating high polydispersity (Figure 1b). TEM was used to inspect the size of bare CQDs and CQDs–AuNPs as well as to analyze the physical association between CQDs and metal nanoparticles. The TEM micrographs of the bare CQD and hybrid prepared via 0.25 mM of HAuCl_4_·3H_2_O are presented in Figure 2a,b. In bare CQD, the average size of the particles is 7 ± 1 nm and single line lattice spacing is measured as 0.26 nm (Figure 2a). In the case of hybrids, two different populations corresponding to CQDs and AuNPs are clearly visible in the TEM micrograph as seen in Figure 2b. Some of the AuNPs bear crystallinity defects, while a minority exhibit single crystalline structures.

**Figure 3:**
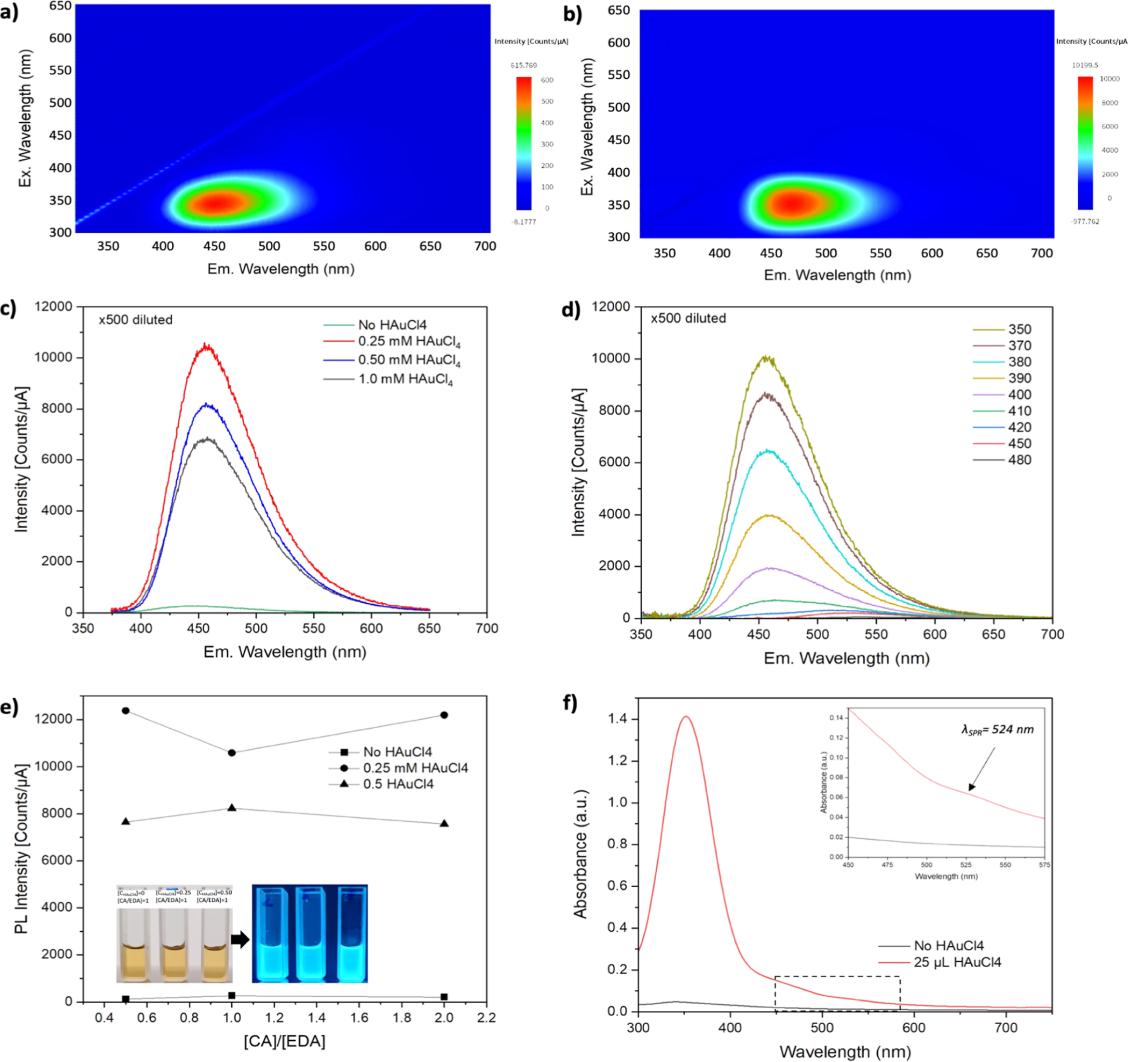
EEMs of CQDs prepared (a) without HAuCl_4_·3H_2_O and (b) with 0.25 mM HAuCl_4_·3H_2_O; (c) fluorescence emission spectrum of CQDs prepared with different concentrations of gold salt; (d) fluorescence emission spectrum of CQDs prepared with 0.25 mM HAuCl_4_·3H_2_O at varying excitation wavelengths; (e) effect of the CA/EDA molar ratio on PL intensity; (f) CQDs and CQDs–AuNPs hybrid absorption spectrum.

As shown in Figure 3a,b, CQDs prepared without gold and with gold (0.25 mM HAuCl_4_·3H_2_O) nearly display similar PL centers in excitation–emission maps (EEM). CQDs exhibit the main emission center in the blue region at 447 nm (Figure 3a), whereas CQDs–AuNPs exhibit a main blue emission center at 457 nm (Figure 3b). The corresponding fluorescence spectra of the CQDs and CQDs–AuNPs are also shown in Figure 3c. The PL intensity is highly affected by the presence of AuNPs in the environment, indicating that the PL intensity increases drastically with the simultaneous formation of AuNPs due to MEF. In this case, the SPR (524 nm), which is quite close to the emission of CQDs at 457 nm, provides a local field enhancement in incident excitation, causing an increase in PL intensity. Whereas a gradual increase of gold salt from 0.25 to 1 mM yields a substantial decrease in PL. The reason for such a decrease could be attributed to the non-stochiometric partition of CA, CQD precursor, and reducing/capping agent for AuNPs during nucleation of CQDs and AuNPs. The nucleation rate of N nanoparticles during time t is described as
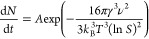
1where *A* is a pre-exponential factor, γ is surface energy, ν is the molar volume of solution, *T* is the temperature, *k*_B_ Boltzmann’s constant, and *S* is the supersaturation of the solution.^27^ During CQD formation, an increase in carbon source concentration up to a supersaturation will also increase the nucleation rate. In the case of hybrid formation, where two nucleation processes occur simultaneously, increasing gold salt concentration will cause a partition of CA between the nucleation of carbon and AuNPs.^28^ The nucleation and growth kinetics of AuNPs are much faster than those of CQD, causing the majority of CA to be used during AuNP formation. For higher gold salt concentrations (>0.25 mM), we have seen black precipitates at the bottom of the reaction flask, indicating the formation of bigger AuNPs (Supporting Information Figure S1).

For the samples prepared using 0.25 mM gold salt, when the excitation wavelength varied from 350 to 480 nm, it was seen that the PL intensity and position were strongly affected by the excitation wavelength, as shown in Figure 3d. When the excitation wavelength increased from 350 to 480 nm, the emission maximum shifted from 457 to 545 nm.

In the hydrothermal synthesis of CQDs, previous works have shown that the concentration of carbon precursors plays an important role in the PL intensity of the final nanomaterial.^29^ In these studies, during CQD growth, higher precursor concentrations lead to higher PL intensity due to the high extent of carbonization. To understand the effect of precursor concentration on PL emission characteristics of CQDs–AuNPs hybrids, different syntheses were realized by varying the molar ratios of CA to EDA (molar ratios CA/EDA 0.5, 1, and 2) at different gold salt concentrations. The results are presented in Figure 3e. It is clear that the PL intensity is not predominantly affected by the CA/EDA ratio, not to the extent that of gold salt. The UV–vis absorption spectrum of CQDs–AuNPs, presented in Figure 3f, reveals a small shoulder around 524 nm corresponding to the SPR peak of AuNP. This peak is not visible in CQDs synthesized without gold salt.

**Figure 4:**
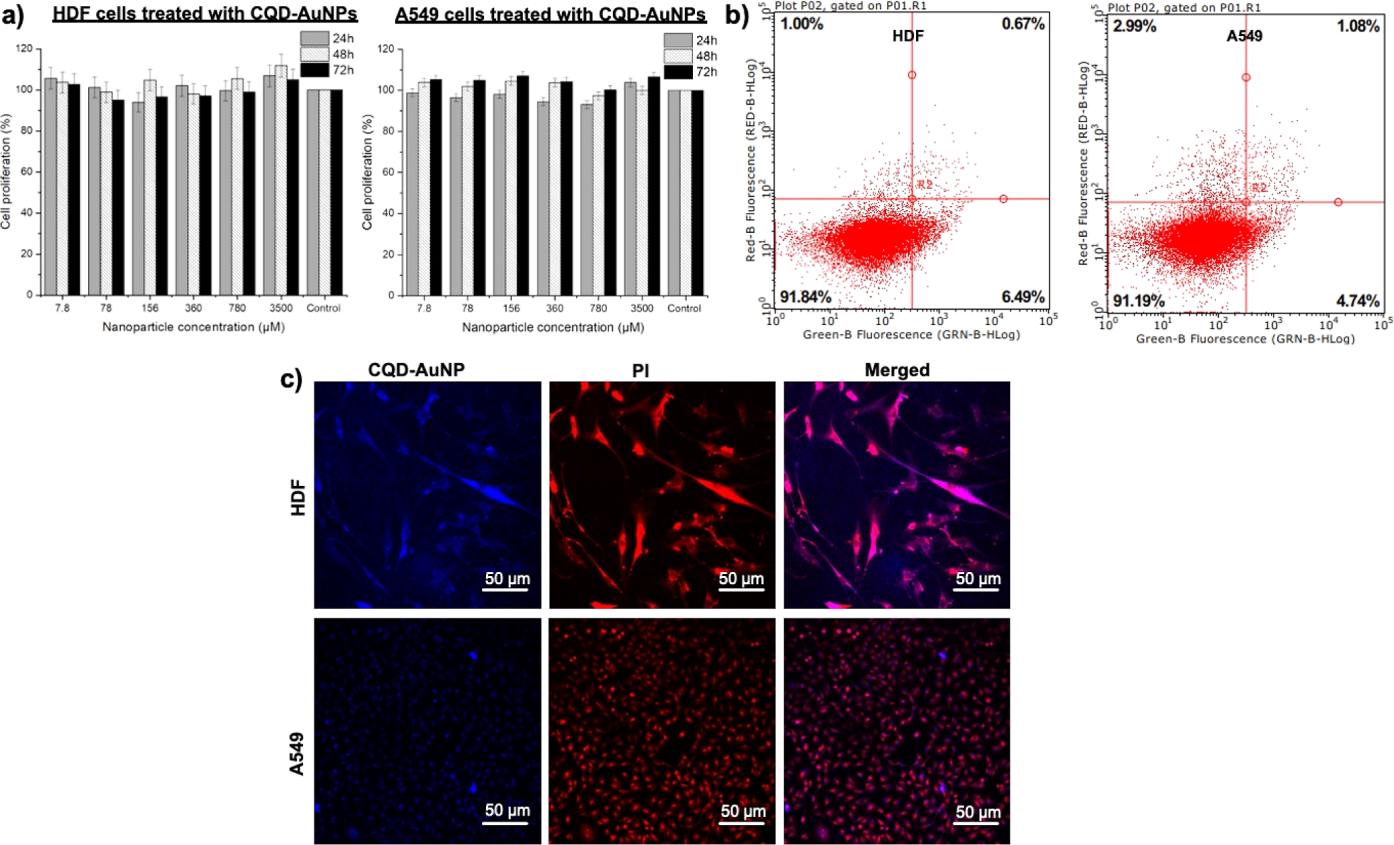
Proliferative and cytotoxic effects of the CQDs–AuNPs with the treatment of HDF and A549. (a) The cells were treated with CQDs–AuNPs, synthesized with 0.25 mM HAuCl_4_, for 24–48 and 72 h in a range of 0–3500 μM, and the proliferative effects of the nanoparticles were investigated via the MTS assay. (b) Cells were treated with 750 μM CQDs–AuNPs for 24 h. After 24 h apoptotic cells were investigated with annexin PI staining. (c) Representative confocal microscopy images of HDF and A549 cells labeled with CQDs–AuNPs, synthesized with 0.25 mM HAuCl_4_.

The cytocompatibility of CQDs–AuNPs was investigated by using cell proliferation and apoptosis assays on HDF and A549 cell lines. During the analysis, both cells were treated with several doses of freshly synthesized CQDs–AuNPs. Figure 4a shows the MTS proliferation effects of CQDs–AuNPs. The results indicate that CQDs–AuNPs does not show any significant proliferative effect compared to the negative control. Similarly, the cellular cytotoxicity effects of CQDs–AuNPs analyzed via annexin–PI indicate that CQDs–AuNPs does not show any cytotoxic activity on healthy or cancer cell lines (Figure 4b).

To verify the staining ability of CQDs–AuNPs, HDF and A549 cell lines, incubated with 750 μL CQDs–AuNPs for 24 h, were analyzed under a laser confocal fluorescence microscope at excitation wavelengths of 405 nm. Figure 4c shows the confocal microscopy images of the cells in comparison with the fluorescent PI cell dye used for DNA labeling.^30,31^ A comparison of the images acquired both for HDF and A549 cells revealed that CQDs–AuNPs are internalized into cells quite well. In merged images, the localization of CQDs–AuNPs with PI gives purple-pink-colored areas. It is also worth mentioning that the application of CQDs–AuNPs is quite simple compared to conventional cell tracker dyes. No pre-optimization is needed, and mixing CQDs–AuNPs with fresh culture medium provides sustainable staining.

## 4 Conclusions

The current study’s findings show that one-pot solid-state synthesis of CQDs with plasmonic AuNPs yields a hybrid material with enhanced PL properties in comparison to its counterparts with no gold present. The presence of gold significantly improves the emission properties of the material due to local field enhancement in incident excitation with SPR.

The CQDs–AuNPs hybrid exhibits high cytocompatibility in both healthy and cancer cell lines. The high staining efficiency of the hybrid illustrates that the material can be used for bioimaging applications. Due to the simplicity of sample preparation, the method is not time-consuming in comparison to traditional cell staining methods.

## Supporting Information Available

Normalized PL emission spectra of the CQDs–AuNPs hybrid and the CQDs, image of CQDs–AuNPs solution, and additional TEM micrographs of CQDs–AuNPs (PDF)
