# Supplementary material for: Metal-Enhanced Fluorescent Carbon Quantum Dots via One-Pot Solid State Synthesis for Cell Imaging
Source: ACS Appl Bio Mater. 2023 Apr 26;6(5):1798–805. doi: 10.1021/acsabm.3c00040 (PMC10189791; doi:10.1021/acsabm.3c00040)
Supplement: Supplementary file 1 — mt3c00040_si_001.pdf [file mt3c00040_si_001.pdf]

Supporting Information

Metal-Enhanced Fluorescent Carbon Quantum Dots via One Pot Solid State Synthesis for Cell Imaging

Volkan Can[1*], Bugra Onat[1], Elif Sümeyye Cirit[2], Fikrettin Sahin[1], Zeliha Cansu Canbek Ozdil[2]

[1]Department of Genetics and Bioengineering, Yeditepe University, Istanbul, Turkey

[2]Department of Materials Science and Nanotechnology Engineering, Yeditepe University, Istanbul, Turkey Abstract

[*]Corresponding author: volkan.can@yeditepe.edu.tr

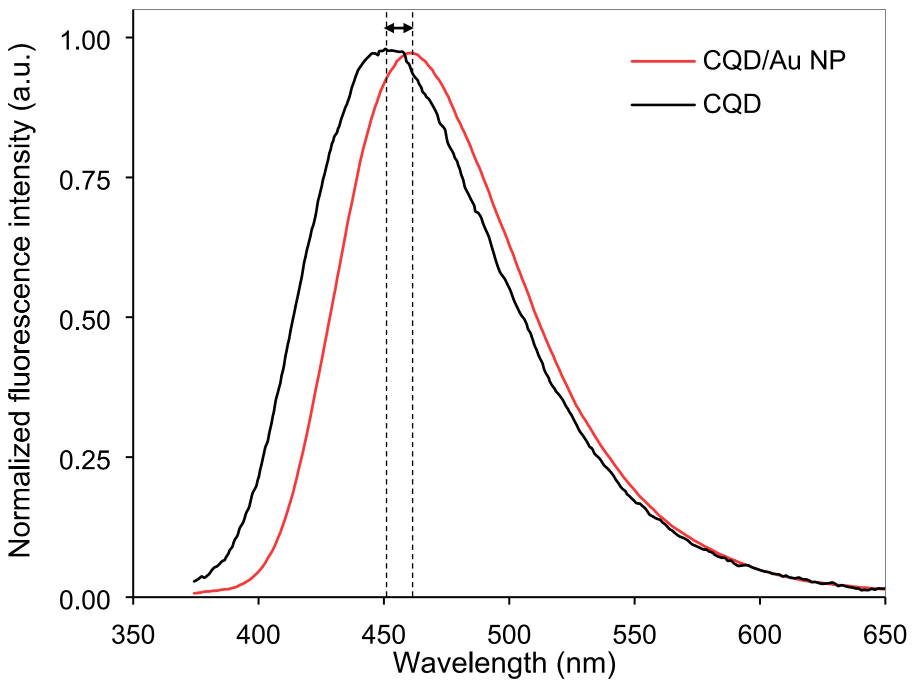

Figure S 1. Normalized photoluminescence emission spectrum of CQD-AuNP hybrid and CQD

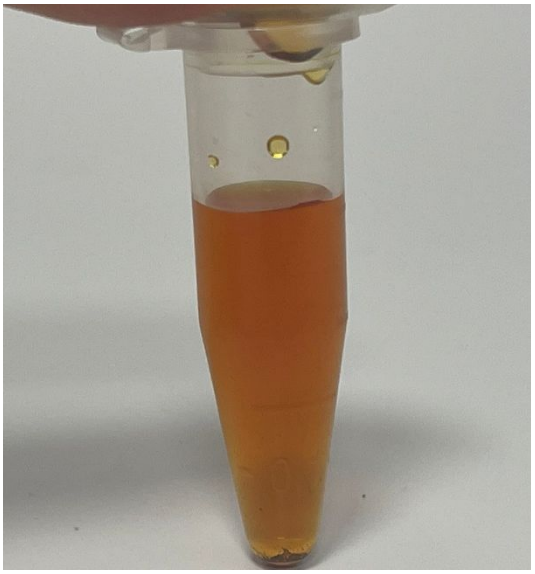

Figure S 2 CQD-Au NP solution prepared with 0.5 mM $HAuCl_4$

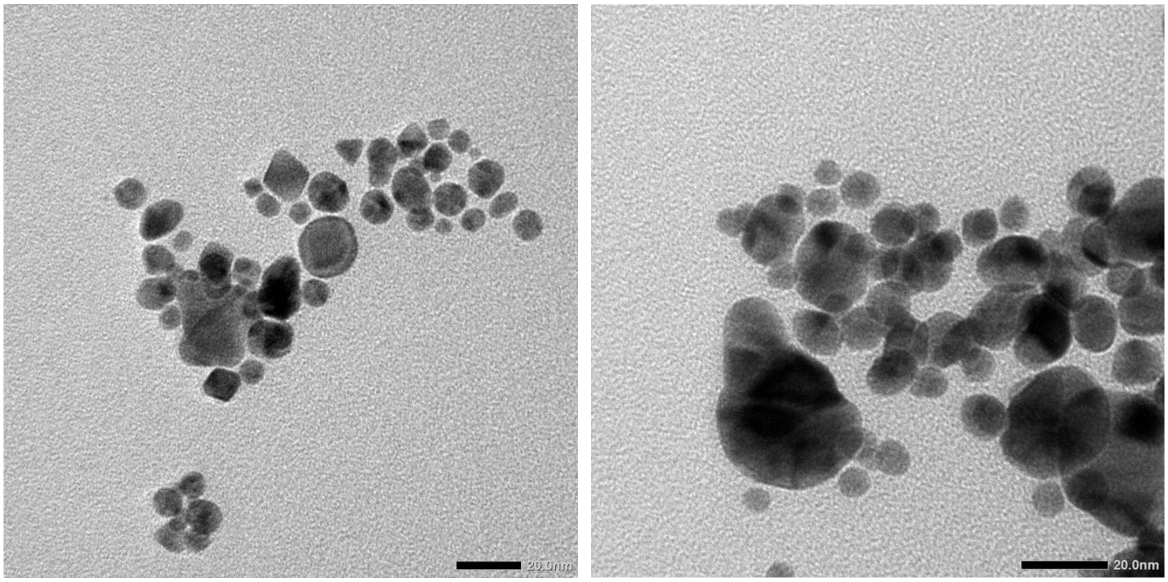

Figure S 3 TEM micrographs of CQD-AuNPs
